# Effect of glass infiltration and modified cooling rates on color characteristics alteration of monochrome and multilayer high yttrium oxide containing zirconia

**DOI:** 10.4317/jced.62066

**Published:** 2024-09-01

**Authors:** Pithiwat Uasuwan, Niwut Juntavee, Apa Juntavee

**Affiliations:** 1 Division of Biomaterials and Prosthodontics, Faculty of Dentistry, Khon Kaen University, Khon Kaen, Thailand; 2 Department of Prosthodontics, Faculty of Dentistry, Khon Kaen University, Khon Kaen, Thailand; 3 Division of Pediatric Dentistry, Department of Preventive Dentistry, Faculty of Dentistry, Khon Kaen University, Khon Kaen, Thailand

## Abstract

**Background:**

Sintering technique impacted color of zirconia. This study examined the effect of glass infiltration and altering cooling rate on color alteration of monochrome (Mo) and multilayer (Mu) 5 mol% yttria-partially stabilized zirconia (5Y-PSZ).

**Material and Methods:**

180 specimens (width, length, thickness = 10, 20, 2 mm) were prepared from Mo and Mu (comprising cervical (C) and incisal (I) zone) 5Y-PSZ shade VITA-A2. Unintentionally categorized samples (n=15/group) were sintered with traditional (T) versus glass infiltrated (G) technique and cooled down with slow (S: 5°C/min), normal (N: 35°C/min), and fast (F: 70°C/min). CIE-L*a*b* color characteristics were determined for translucency parameter (TP), contrast ratio (CR), opalescence parameter (OP), and color difference (∆Ediff). Microstructures were investigated with SEM and XRD. ANOVA and Tamhane’s comparisons were determined for significant differences (*p*<0.05).

**Results:**

TP and OP were significantly higher for Mo than MuC and MuI, but no significant difference in CR among them. Comparable ΔEdiff between Mo and MuC were indicated, but both were significantly lesser than MuL. Glass infiltration and raising cooling rate significantly decreased TP and OP, whereas increased CR and ΔEdiff, which amplified color alteration.

**Conclusions:**

Glass infiltration sintering and modified cooling rate significantly altered color parameters of 5Y-PSZ. Monochrome demonstrated higher translucency and opalescence than multilayer, possibly due to colorant additives. Glass infiltration decreased translucency and opalescence due to different refractive indices. Increased cooling rate resulted in decreasing translucency and opalescence due to smaller grain size and t→m transformation. Nevertheless, altered sintering and cooling rates still rendered an acceptable color alteration.

** Key words:**Cooling rate, glass infiltration, optical characteristics, translucency.

## Introduction

Dental ceramics have been utilized for esthetic applications for many years due to their exceptional physical characteristics. As a consequence of the ongoing development of digital technology, several state-of-the-art dental ceramics have been introduced in response to patient needs. One of the appealing ceramics is stabilized zirconia, due to its excellent physical and mechanical properties, dimensional stability, and chemical inertia (1). Zirconia encompassed three microstructural phases: monoclinic (m-), tetragonal (t-), and cubic (c-) phases. These phases are interchangeable due to the triggered temperature. At the ambient temperature, the m-phase was detecTable. Upon heating up to 1,170ºC, the m-phase was induced to transform to the t-phase until the temperature reached 2,370ºC, and the c-phase appeared and remained unchanged up to the melting point of 2,680ºC (2). As cooling down, all the microstructures turn into the m-phase. To achieve and stabilize the desired t- and c-phases at room temperature, a yttrium oxide stabilizer (Y2O3) was added (2). The 3 mol% yttrium oxide was first implemented into zirconia as called 3 mol% yttria-stabilized tetragonal polycrystalline (3Y-TZP). This 3Y-TZP presented almost the entire t-phase at the normal temperature. The zirconia microstructure transitions from the t- to m-phase causing 4–5% expansion as induced by external stimuli such as moisture, stress, and warmth, giving stabilized zirconia an exceptional strength called “transformation toughening”, a process that creates compressive stress generated by the volumetric expansion of phases to withstand crack propagation (1). However, the light scattering on 3Y-TZP, given its opacity, has to be tremendously concerned for monolithic use.

A variety of techniques have been employed to enhance the light transmission and translucency of monolithic zirconia, such as varying the sinter settings, adjusting the quantity and size of alumina, and altering the yttrium oxide concentration (3). Adding up to 5 mol% yttrium oxide to monolithic zirconia, called “5 mol% yttria-partially stabilized zirconia (5Y-PSZ)”, can develop approximately 50% cubic structures (3). Because of their larger volume and increased isotropy, these cubic structures improve material translucency by reducing light scattering at the grain boundaries and uniformly radiating incident light across the structure (3). However, this extremely translucent 5Y-PSZ ceramics presented with a decreased mechanical strength since it had less transformation toughening as a result of the minimal t-phase presented (4). Monolithic zirconia can be classified into monochrome (Mo) and multilayer (Mu) zirconia. The Mo zirconia represents the single material’s color for the entire zirconia block, while the Mu zirconia has a chromatic gradient shift from the cervical to the incisal area mimicking the natural dentition (5). Previous studies proved that the color gradience between each zone of Mu zirconia was due to the pigment addition (5). For example, increasing ferric oxide (Fe2O3) led to a material loss of translucency (6). The utilization of increased cerium and bismuth concentrations led to a significant rise in the green-blue (b*) color coordinate (7). However, certain studies indicated that there was no significant difference in translucency (5) and flexural strength (8) across different layers of zirconia. The Mo and Mu 5Y-PSZ are mostly limited to use for single and short-span fixed restoration due to their sacrificed strength, and the translucency was still lesser than that of lithium disilicate glass ceramics (9). When choosing a material, especially in the realm of aesthetics, optical qualities such as translucency, contrast, opalescence, and color perception are the most important factors to take into account (10). The amount of light transmission through an item was used to characterize its translucency. Translucency increases with increasing light transmission and is usually assessed by translucency parameter (TP) and contrast ratio (CR) (11). The substance exhibiting increased translucency essentially possesses a higher TP value and a reduced CR value (11). In addition, the light trajectory has been observed to be influenced by the material’s grain size and distribution, crystal microstructure, color pigments, and defect pores (11). The process is when light is reflected off the object, it should seem blueish, and when light passes through it, it should appear orange, was called opalescence. It is vital for accurately imitating the natural enamel and can be determined by the opalescence parameter (OP) (12). The OP value of natural enamel of 19.80–27.60 was reported (13). The perception of color alteration is related to the color perceptibility threshold, which was investigated through the color difference value (∆Ediff). This value could be classified as within the perceptibility threshold (PT, ∆Ediff ≤2.6) and the acceptability threshold (AT, ∆Ediff ≤5.5) (14). The more the color difference value, the more the color distinguishment, and the less the acceptability in color alteration.

An attempt to improve the strength and esthetics of 5Y-PSZ is to invent a suiTable silicate glass with a lower modulus to be graded on the outer surfaces of porous zirconia as called graded zirconia (15,16). Such a gradation is expected to reduce the tensile stress intensity at the outer surfaces of restoration (17), where fractures frequently begin, making the structure less prone to fracture. In terms of esthetics, a study showed that the translucency of the infiltrated zirconia was improved relatively to those of conventional core materials; however, it was not as high as that for dental porcelains (18). On the other hand, another study showed a decreased translucency and increased color difference value than the non-infiltration counterpart (19). The glass infiltration can be done on intaglio and/or cameo surfaces of restoration. The glass deposition on the external surface also acts as an encapsulation layer that could oppose water absorption, prevent hydrothermal degradation, protect against antagonist attrition, and permit color disparities, eventually leading to esthetics improvement (15,20). Moreover, the formation of glass on the cementation surface makes acid etching and silanization feasible, enhancing bonding to resin cement (20). The color of the glass infiltration could be adjusted to match the surrounding structure by incorporating color additives during the process of staining after the glass infiltration (16). This glass infiltration requires a first firing procedure for making more stabilized porous zirconia from the pre-sintered block and a second firing procedure for the glass infiltration into the porous zirconia. Therefore, the fabrication of graded zirconia is time-consuming.

To improve the physical properties of stabilized zirconia, the adjustment of firing parameters was considered a cost-effective strategy. This modification impacted material density, pore reduction, and crystal structure (21). Firing monolithic zirconia at higher temperatures was known to increase its translucency (21). Additionally, prolonging the sintering time of 3Y-TZP led to grain growth, significantly improving optical characteristics and triggering the t- to m-phase transition (10). Rapid cooling of stabilized zirconia has been shown to increase translucency by promoting bigger grain size (22) and inducing a t- to m-phase change (23,24) The impact of altering cooling parameters on color characteristics were still insufficient, and controversial results regarding glass infiltration in 5Y-PSZ. Until now, there was no commercial product for glass infiltration with suiTable firing parameters to optimize the physical characteristics of dental zirconia. The invention of glass composition has been challenging for researchers and requires further experimental tests. In this study, we developed glass infiltration powder along with different sintering techniques and examined their impacts on the alteration of color characteristics of Mo and Mu 5Y-PSZ. The null hypothesis of this study postulated that the color characteristics of 5Y-PSZ would not be influenced by the different sintering techniques based on glass infiltration, cooling rates, types of materials, and their interactions.

## Material and Methods

**Figure 1:**
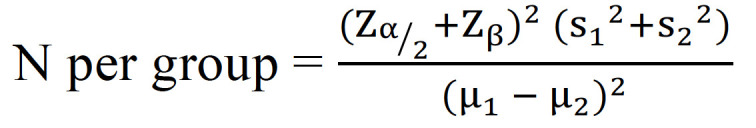
Equation 1.

The sample size for this experimental study was derived from the statistical data performed by Sailer and colleagues’ publication in 2007 (25) using G*power 3.1 software (Heinrich-Heine-Universität, Düsseldorf, Germany) with a power of test = 0.90 and α error = 0.05 as shown in Equation 1, (Fig. 1):

*Where: Zα = standard normal deviation = 1.96 (α error = 0.05), Zβ = standard normal deviation = 1.28 (β error = 0.1), µ1 - µ2 = mean difference between tested group = 0.8, and s = standard deviation (s1 = 2.3, s2 = 1.5). The sample size was calculated and employed 15 specimens per group.*

-Preparation zirconia specimens

One hundred eighty (180) tested specimens were prepared from monochrome (Mo: Cercon xt, Dentsply Sirona, Charlotte, NC, USA), and multilayer (Mu: Cercon xt ML, Dentsply Sirona) 5Y-PSZ block. The chemical composition of each 5Y-PSZ is shown in  Table 1. The Mo and Mu 5Y-PSZ samples were cut with a diamond-coated wheel at a speed of 700 rounds per minute (rpm) by a micro-cutting machine (Mecatome T180, PRESI, Eybens, France) with water coolant. Waterproof SiC abrasive sheets up to no. 2000 were used to grind the 5Y-PSZ samples in a wet condition with water using a grinding machine (Ecomet 3, Beuhler, Lake Bluff, IL, USA) at 40 rpm speed. After that, specimens were polished using suspension with fine diamond particles mounted to a polishing apparatus (Ecomet 3, Beuhler) at 80 rpm speed. The materials were decontaminated for ten minutes with an ultrasonic cleanser (Vitasonic II, Vita Zahnfabrik, Bad Säckingen, Germany) in distilled water and finally dried for an hour at room temperature. To attain a specified size (width x length x thick = 10 x 20 x 2 mm), the samples were originally prepared to an enlarged bar (width x length x thick = 12.5 x 25 x 2.5 mm) for compensation of sintering shrinkage. After the sintering was completed, the zirconia specimens achieved their final dimensions and were measured using digital vernier calipers (Mitutoyo, Kanagawa, Japan).

-Preparing glass infiltration powder

The glass infiltration powder (In-house glass, Division of biomaterials, Khon Kaen University, Khon Kaen, Thailand) was prepared as its elemental composition was shown in  Table 1. These oxides were ground and uniformly mixed in a crucible. Finally, a serial sieving procedure was done in a chamber to achieve a fine powder size under 150 mesh. The glass infiltration powder was stored in a sealed glass bottle before use.

-Zirconia sintering procedure

The Mo and Mu 5Y-PSZ specimens were randomly distributed to be sintered according to two sintering techniques: glass infiltration (G), and traditional (T) sintering, and three different cooling strategies: slow cooling rate (S) at 5°C/min, normal cooling rate (N) at 35°C/min, and fast cooling rate (F) at 70°C/min (n = 15/group). For G-sintering groups, the 5Y-PSZ specimens were initially sintered to form the porous zirconia by heating to 880°C at a speed of 22°C/min, then 11 °C/min to reach a maximum temperature of 1350°C and held for 1 hour, followed by cooling down per three cooling rate strategies (S, N, and F). After reaching room temperature, the in-house glass was mixed with distilled water and then applied to the surface of the zirconia specimen with a soft camel brush. The 100-micron thick glass was applied on one external surface side and was controlled by a custom acrylic template. A final sintering was done by heating to 880°C at a speed of 22°C/min, then 11°C/min to reach a maximum temperature of 1500°C and held for 2 hours. After that, cooling down to normal temperature using the assigned cooling speeds. (S, N, and F). For T-sintering groups, the 5Y-PSZ specimens were sintered by heating to 880°C at a speed of 22°C/min, then 11°C/min to reach a maximum temperature of 1500°C and held for 2 hours. After that, cooling down to normal temperature using the assigned cooling speeds. (S, N, and F). The aforementioned sintering procedure was performed in a high-temperature furnace (inFireHTC, Dentsply Sirona). The sintered specimens were cleaned by a high-frequency ultrasonic device (Vitasonic II, Vita Zahnfabrik) for 10 minutes, and were allowed to dry at room temperature before testing.

-Determination color characteristics 

**Figure 2:**
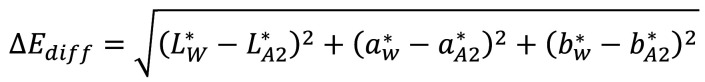
Equation 2.

The color characteristics of traditional and glass-infiltrated Mo and Mu 5Y-PSZ with different sintering strategies were achieved using a laboratory spectrophotometer (ColorQuest XE, Hunter, Reston, VA, USA). A transparent acrylic template was employed to keep the specimen in place. The cervical (C) and incisal (I) portions of Mu 5Y-PSZ were measured independently. The testing apparatus was adjusted to illuminant D65, 10% observer angle, 100% UV, and a standard wavelength of 380-780 nm. The determinations were made in CIE1976 (Commission Internationale de I’Eclairage). The L*, a*, and b* parameters were attained from the lightness, the green-red coordinate, and the blue-yellow coordinate of specimens, respectively, on black (B) (LB* = 10.40, aB* = 0.40, bB* = 0.60), and white (W) background (LW* = 96.70, aw* = 0.10, bw* = 0.20). Then, the translucency parameter (TP), contrast ratio (CR), opalescence parameter (OP), and color difference (∆Ediff) were computed by Equation (2-6) (11). The coordinates of the A2 shade of VITA classical (Vita Zahnfabrik) on a white background (LA2* = 65.61, aA2* = -0.50, bA2* = 5.54) were measured and used for determination for the amount of color alteration through ∆Ediff values according to Equation 2 (11), (Fig. 2).

**Figure 3:**
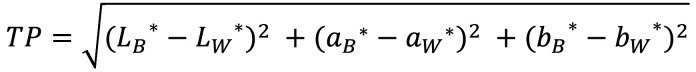
Equation 3.

The translucency was determined from the TP values that were calculated from the differences between color determinants on black (B) and white (W) backgrounds, according to Equation 3 (11), (Fig. 3):

The contrast was determined from the CR values using Equations 4 and 5 in which the CR values range from 0.00 (transparent) to 1.00 (perfectly opaque) (11). In terms of Tristimulus Color Space, Y represents the brightness illuminance; YB and Yw are the values of a sample placed on the black and white backgrounds, respectively; and Yn is equal to 100, (4,5):

**Figure 4:**
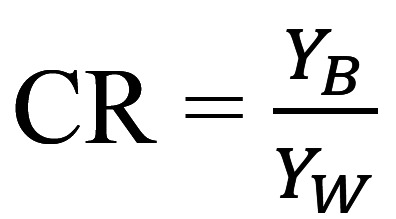
Equation 4.

**Figure 5:**
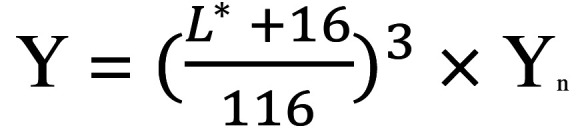
Equation 5.

**Figure 6:**
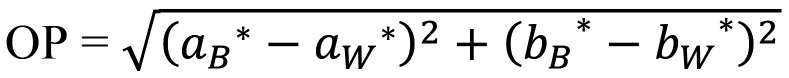
Equation 6.

The opalescence was determined from the OP values that were achieved by using Equation (6,11), (Fig. 6):

-Determining the microstructure and chemical composition

Three specimens from each group were randomly selected for electroplating with gold for three minutes at 10 mA of a current and a vacuum of 130 mTorr and then were dried in a desiccator cabinet. For the Mu 5Y-PSZ, the cervical (C) and incisal (I) zones were evaluated separately. A scanning electron microscope (SEM, Hitachi SU3800, Osaka, Japan) coupled with an energy dispersive spectroscopy (EDS) were used to evaluate the microstructure and chemical components. The software (Image J, U.S. National Institutes of Health, Bethesda, MD, USA) was used for measuring the grain size. The cross-sectional micrograph was also performed to determine the characteristics of residual glass, and infiltration zone.

-Determining the phase proportion

**Figure 7:**
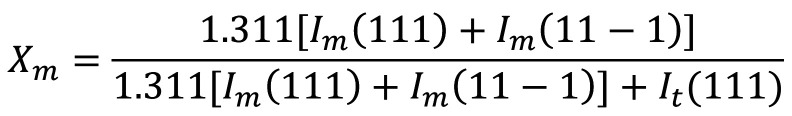
Equation 7.

**Figure 8:**
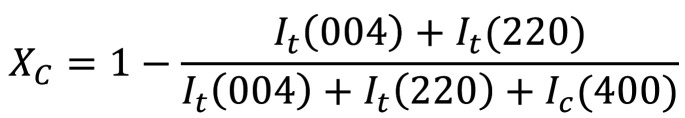
Equation 8.

**Figure 9:**
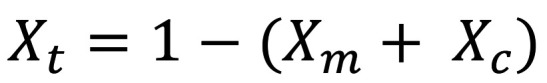
Equation 9.

The relative proportions of zirconia crystal phases were measured using an X-ray diffractometer (MiniFlex 2, Rigaku, Tokyo, Japan). The specimens were scanned with copper k-alpha (Cu Kα) radiation at intervals of two seconds, using diffraction angles (2θ) ranging from 20 to 80 degrees with a step of 0.02o. The zirconia phase was evaluated by cross-referencing the Joint Committee of Powder Diffraction Standards database file (PDF). The analysis of the m-, t-, and c-phase proportion was performed by Rietveld refinements with powder diffraction analysis software (Match! software version 3.15, Bonn, Germany) based on the peak intensity. The peaks were referenced based on PDFs No. 37–1484, 49–1642, and 42–1164, for the m-, t-, and c-phases, respectively. The proportion of the m-phase (Xm) was estimated using Equation 7 (24). The Ic, It, and Im, displayed the integrated intensities for the c-, t-, and m-phases, respectively. These were calculated by fitting a pseudo-Voigt distribution to the complimentary peaks and analyzing the area under the curves. To take the impact of yttria doping on the lattice parameters into consideration, a correction factor of 1.311 was determined using the non-linear calibration curve of integrated intensity ratios versus volume fraction. The proportion of t-phase (Xt) and c-phase (Xc) was calculated from Equations 8 and 9 (24), (Figs. 7,8,9).

-Statistical analysis

The data were executed with the Shapiro-Wilk test for normality test, and Levene’s test for homoscedasticity test using statistical software (IBM SPSS V-28, SPSS, Chicago, IL, USA). Since the data were normally distributed and presented homoscedasticity (*p*>0.05), the three-way ANOVA and Post hoc Tamhane-T2 multiple comparisons were performed to detect substantial variations in the optical characteristics between Mo and Mu 5Y-PSZ under the influence of different sintering techniques and various cooling protocols. A statistically significant difference was set at *p*<0.05. In addition, descriptive analysis was employed to assess the optical properties, grain size, elemental composition, and relative phases of the zirconia.

## Results

**Figure 10:**
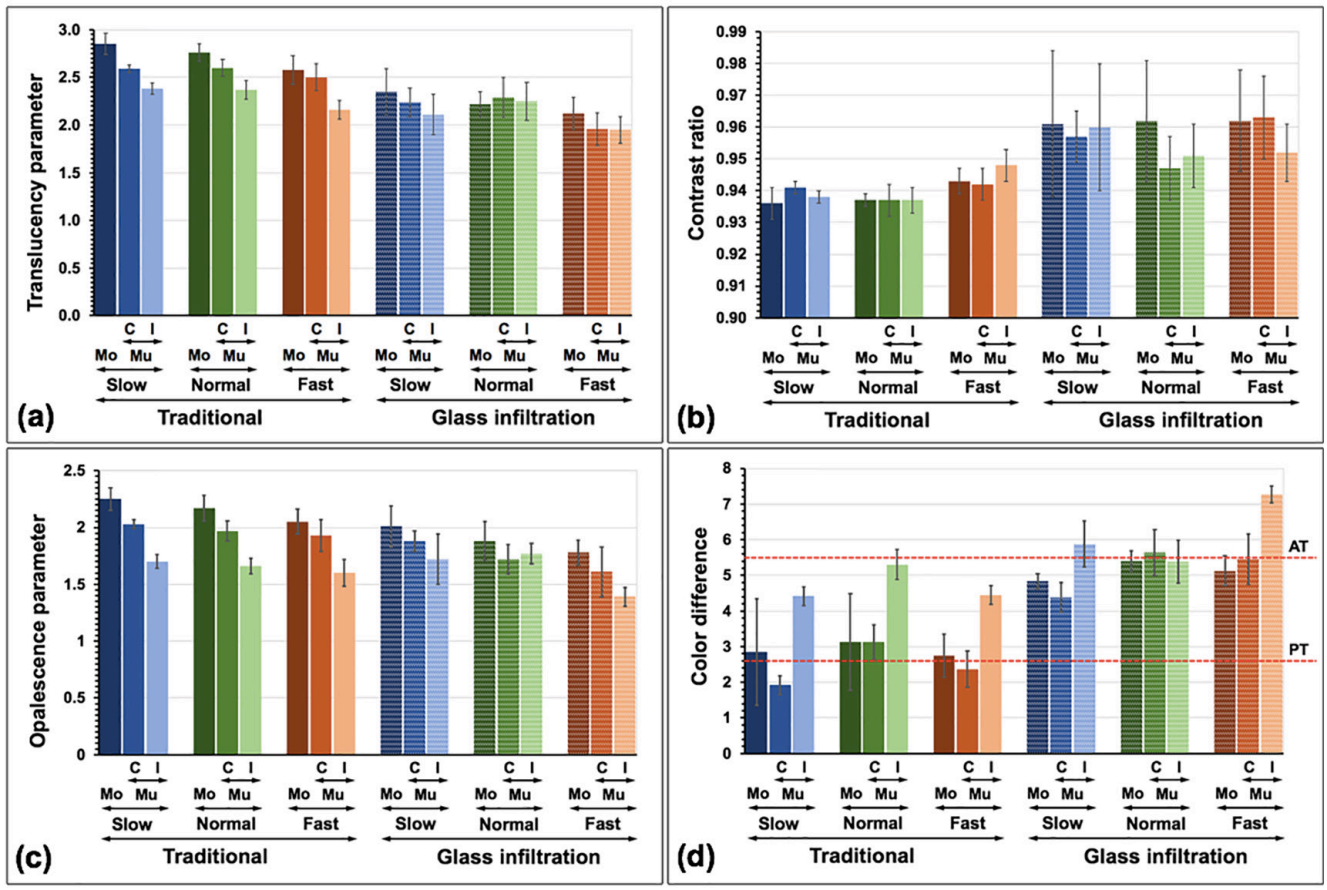
Translucency parameter (a), contrast ratio (f), opalescence parameter (c), and color difference (d) with perceptible threshold (PT) and acceptable threshold (AT) of traditional and glass infiltrated monochrome (Mo), multilayer (Mu, C: cervical; I: incisal) 5Y-PSZ upon slow, normal, and fast cooling rate were shown.

**Figure 11:**
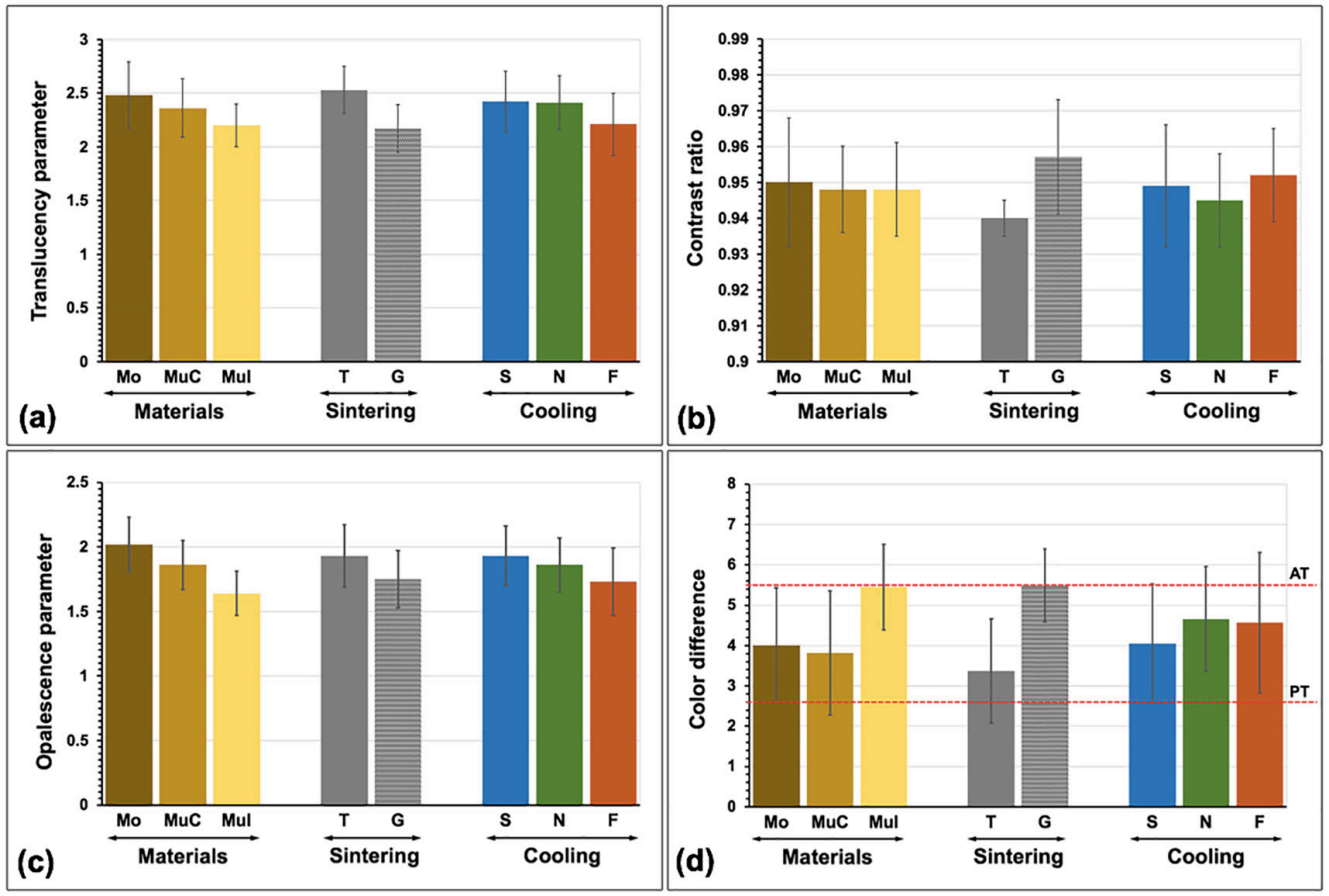
Influence of materials [monochrome (Mo), multilayer (Mu, C: cervical; I: incisal)], sintering techniques [traditional (T), glass infiltration (G)], and cooling rates [slow (S), normal (N), fast (F)] on translucency parameter (a), contrast ratio (b), opalescence parameter (c), and color difference (d) with perceptible (PT) and acceptable threshold (AT).

The mean translucency parameter (TP), contrast ratio (CR), opalescence parameter (OP), and color difference (ΔEdiff), along with their standard deviation (SD) of experimental groups were presented (Fig. 10 and ( Table 2). The type of zirconia materials, sintering strategies, cooling rates, and their interactions significantly affected all color characteristic values (*p*<0.05), except for material to CR, the interaction of material and cooling to CR, and the interaction of sintering and cooling to TP and CR, as revealed by the result of three-way ANOVA ( Table 3). Post hoc Tamhane-T2 results for each optical parameter were presented in Table 4. Regarding the Mu 5Y-PSZ materials, MuC presented significantly higher TP, and OP but lower ΔEdiff than MuI (*p*<0.05). The Mo demonstrated closer ΔEdiff values to MuC than to MuI. The Mo exhibited the highest translucency and opalescence, followed by MuC and MuI, respectively. Increasing the cooling rate and/or infiltration with the glass significantly increased in CR, and ΔEdiff whereas significantly decreased in TP, and OP values (*p*<0.05) as presented in Post hoc multiple comparisons ( Table 4). The application of glass and increasing the cooling rate produced a more white, less red-yellow, and more green-blue appearance in 5Y-PSZ (Fig. 11). Concerning the color alteration compared to A2 VITA Classical shade (ΔEdiff), the MuCS and MuCF were considered within a PT (ΔEdiff ≤ 2.6), whereas the remaining groups except GMuIS, GMuCN, and GMuIF were considered within an AT (ΔEdiff ≤ 5.5). However, the mean ΔEdiff value of the different types of materials (Mo, MuC, and MuI), sintering techniques (T, and G), or different cooling rates (S, N, and F) was within the AT.

**Figure 12:**
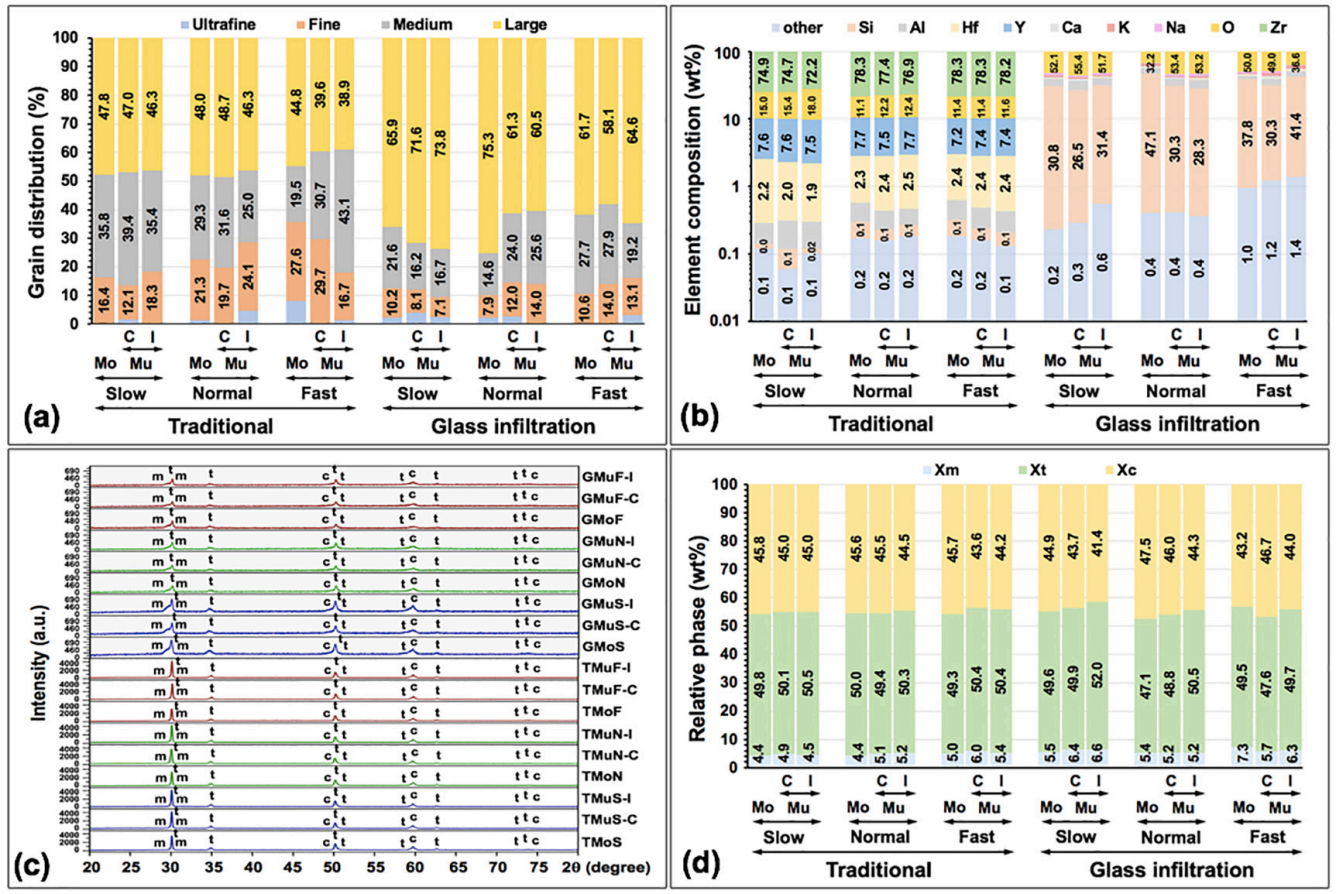
Grain distribution (a), elemental composition (b), x-ray diffraction angle (c), and relative phase of zirconia (d) of traditional (T) and glass infiltrated (G) monochrome (Mo) and multilayer [Mu, at cervical (C), and incisal (I) layer] 5Y-PSZ upon slow- (S), normal- (N), and fast- (F) cooling protocols.

**Figure 13:**
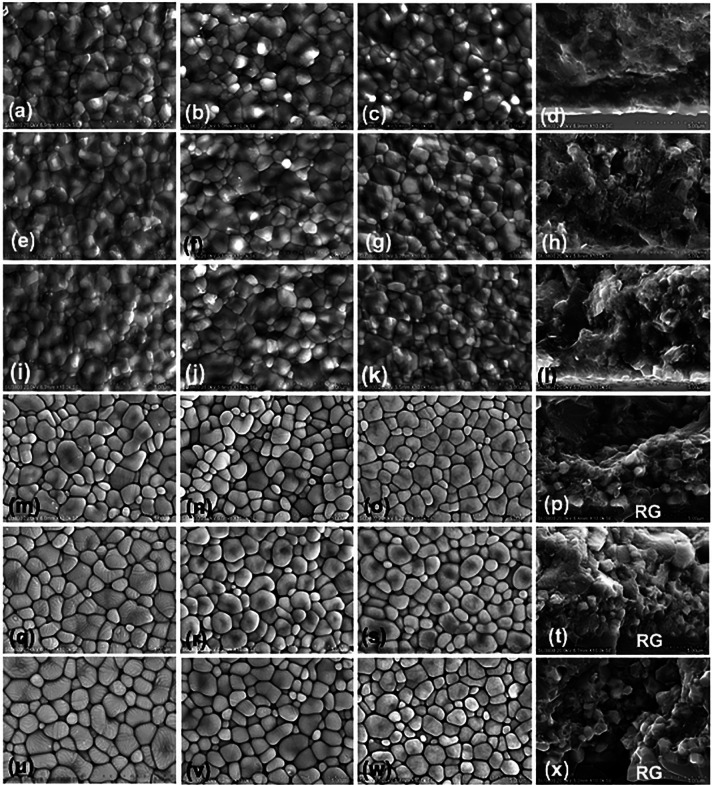
Scanning electron microscope photomicrographs at ×10K magnification indicated grain size and grain distribution of 5Y-PSZ traditional monochrome (a-d), multilayer [E-L: cervical (e-h), incisal (i-l)], and glass infiltrated monochrome (m-p), multilayer [q-x: cervical (q-t), incisal (u-x)], upon slow (a, e, m, q, u), normal (b, f, j, n, r, v), and fast (c, g, k, o, s, w) cooling protocol. The micrographs of the cross-sectional surface of traditional sintered (d, h, l) and glass infiltrated (p, t, x) 5Y-PSZ, revealed a layer of residual glass (RG).

The microscopic structures of Mo and Mu 5Y-TZP were depicted from the SEM and EDS. The zirconia crystals were classified into four groups based on their grain size: ultrafine (≤0.4 µm), fine (0.4<x≤0.7 µm), medium (0.7<x≤1 µm), and large (>1 µm), and their relative percentage of grain distribution were presented (Fig. 12a,  Table 2). The average grain size (mean±SD, µm) of Mo (1.03±0.42) was larger than MuC (1.02±0.36) and MuI (1.01±0.44). The larger zirconia grain size was observed in glass-infiltrated sintering (1.18±0.42) compared to traditional sintering (1.02±0.40) of 5Y-PSZ. Moreover, the S-cooling protocol illustrated the biggest grain size, followed by the N- and F-cooling protocols. However, the average grain sizes of Mo 5Y-PSZ and C- and I-halves of Mu 5Y-PSZ were not remarkably different (Fig. 13). The relative percentage of the grain size distribution for each type of 5Y-PSZ was influenced by the sintering technique and cooling rate (Fig. 12a,  Table 2). The percentage of large grain sizes was likely to increase while sintering with the glass infiltration technique as well as slowing the cooling rate. Regarding the composition analysis by EDS, the traditional 5Y-PSZ presented major elements (%wt) of zirconia (72.2 - 78.3), oxygen (11.1 - 18.0), and yttrium (7.2 - 7.7). The amount of yttrium was equivalent to 5mol% yttrium oxide. While the amount (%wt) of oxygen (32.2 - 55.5), silica (26.5 - 47.1), and alumina (5-9.8) were detected as a majority on the residual glass of glass infiltrated 5Y-PSZ (Fig. 12b).

The amount of the zirconia phase was analyzed by XRD as illustrated (Fig. 12c,d,  Table 2). The X-ray diffraction patterns of all testing groups depicted the t-phase as the main phase, followed by the c- and m-phase. The t-phase was majorly observed at diffraction angles of 30.00º, 34.86º, 73.20º, and 74.20º, the c-phase was shown at 74.70º, as well as the m-phase was detected at 28.00º and 31.20º. All testing groups showed that the percentage (%) of t-phase (47.1 - 52.0) was slightly higher than that of c-phase (41.4 - 47.5). The number of zirconia phases was correlated to the zirconia sintering technique and the cooling rate. It was observed that the m-phase was likely to increase as a result of infiltration sintering with the glass and fast cooling rate.

The SEM image of traditional 5Y-PSZ, and glass-infiltrated 5Y-PSZ was shown (Fig. 13). The F-cooling pattern impeded the formation of zirconia grains, resulting in smaller grains than those generated by S- and N-cooling patterns in both traditional and glass-infiltrated 5Y-PSZ (Fig. 13). Concerning the surface morphology of the infiltrated zone of glass-infiltrated 5Y-PSZ, the images revealed that the zirconia grains were loosely bonded, and there were gaps between the grains, providing a room for silica infiltration. The SEM cross-sectional image along with the EDS line scan of glass infiltrated 5Y-PSZ illustrated the residual glass of ~60-80 µm, the infiltration area of ~52-59 µm, and the innermost dense zirconia grain. The S-cooling rate seemed to have a lesser infiltration depth and presented thicker residual glass.

## Discussion

In this study, we innovated a glass powder for conducting glass infiltration on Mo and Mu 5Y-PSZ while simultaneously modifying the sintering technique. Both Mo and Mu 5PSZ specimens were firstly fired to create the porous zirconia, which had the gap and inter-grain hollow, and afterward the invented glass onto the surface. Upon the second firing, the glass then infiltrated into a space between grains of porous zirconia by the capillary force, to completely form the inter-networking between glass and zirconia, along with the optimization of sintering cooling strategies, to achieve the glass infiltrated 5Y-PSZ. Subsequently, the color characteristics were measured in comparison between glass infiltrated and traditional (non-infiltration) sintering counterparts. The results revealed significant effects of material type, cooling rates, and sintering strategies, as well as their interactions, on alteration of all color characteristics values (*p*<0.05), except the material to CR, the interaction of material and cooling to CR, and the interaction of glass and cooling to TP and CR. Therefore, the null hypothesis was partially rejected.

Regarding the type of materials, Mo, MuC, and MuI 5Y-PSZ showed significant differences in all parameters except for CR. A slight disparity in color additives, as noted in a previous study (5), played a crucial role in color perception. In the non-infiltration groups, Mo tended to appear whiter, compared to Mu. Additionally, MuI appeared darker, compared to MuC, consistent with the natural character of a tooth where the incisal part typically displays a black incisal halo and more shadowing than the cervical part. The MuC exhibited higher chroma than MuI, resembling the chromatic properties of a natural tooth that increased from I- to C-zone (26). The I-zone had less red-yellow but more green-blue than the C-zone of 5Y-SPZ, possibly due to differences in the addition of color pigments such as Fe (producing green) and Co (producing blue). Moreover, cerium and bismuth in higher concentrations could raise the green-blue coordinate (7). The translucency and opalescence of Mo were significantly higher than MuC and MuI, as observed in TP values, possibly due to differences in grain size. The average grain size of Mo was the largest followed by MuC and MuI. The larger grain allowed more light transmission, leading to more translucency since it presented less grain boundary where the light usually scatters. Another study also supported that the I-zone had less translucency than the C-zone of 5Y-PSZ (26). However, a different study argued that higher TP values were observed in the I-zone instead of the C-zone (27), possibly due to variations in material brand and composition. The different color additives between layers of 5Y-PSZ also affected the translucency of the materials (28). The XRD analysis indicated that the proportion of the c-phase appeared slightly higher in Mo than in MuC and MuI. The isotropic c-phase improved material translucency by exhibiting a more uniform emission of incident light across the structure and causing less light scattering (3).

The application of glass also had significant effects on all color parameters. A more white-chalky appearance and less chroma (less red-yellow but more green-blue) were observed in the glass infiltration group. This color shift resulted in 5Y-PSZ having less translucency, as indicated by reduced TP and raised CR value. This might be attributed to the glass infiltration powder being composed of various elements with different refractive indices, causing light scattering at the boundary between each composition. In the firing process, liquefied glass infiltrated the zirconia grain boundaries, facilitated by capillary action, leading to the formation of a glass-rich layer and a glossy surface (15). The presence of porosity in this glass layer could generate light scattering and reduce TP values, as supported by a previous study (19). On the other hand, another study demonstrated an equal TP value in graded zirconia compared to its non-infiltration counterpart (16). This discrepancy between studies may arise because, in that study, they removed the excessive glass layer from the external surface before conducting optical property tests. In contrast, in our study, we examined the color parameters considering the residual glass thickness of about 60-80 µm. Regarding the microstructure, the grain size of glass-infiltrated 5Y-PSZ increased by approximately 16% compared to the non-infiltration groups. This increase could be attributed to the additional firing procedure performed for glass infiltration, which promoted grain growth and complete integration between grains and into the glass. This enlargement of grain might induce spontaneous transformation from the t- to m-phase upon cooling (23). Such a shift typically occurs when grain sizes are above 1 μm (23). Larger grain sizes generally result in better translucency. However, the graded zirconia, composed of different refractive indices and composition in the residual glass layer, infiltration zone, and dense zirconia, had a more significant influence on light transmission than the overall grain size.

The microstructure of zirconia is influenced by firing settings in various ways, including vacancy, crystalline phase, size and development of the grains, and density (10,21,23,24). In this study, fast cooling significantly rendered the 5Y-PSZ a chalky white and less chromatic (less red-yellow but more green-blue). Fast cooling also led to reduced translucency, as indicated by a decreased TP and an increased CR value. The smaller grain size produced by rapid cooling resulted in more light scattering and less light transmission at the grain boundary, contributing to the decreased translucency of the materials. Rapid cooling could also induce defects or porosity, preventing the complete integration and tight bond of grains (29). However, another study showed better translucency and larger grains in rapid cooling (23); this difference might be attributed to the use of different materials. We used 5Y-PSZ, which had a different yttria dopant, a different amount of zirconia phase, and a different effect on grain size compared to 3Y-TZP in their study. Additionally, we found that the m-phase relatively increased with an increase in cooling speed, supported by a previous study (23). When a material is rapidly cooled, it may experience thermal shock, leading to the formation of residual stresses that induce the microstructural transition of zirconia from the t- to m-phase (10,23,24). The increase in the m-phase might have a more pronounced effect on birefringence among interfaces of different crystal structures. Moreover, the c-phase seemed to decline in fast cooling, which also diminished the translucency of the materials.

Regarding the assessment of the opalescence phenomenon using OP, the aim was to use substances with an OP value that closely resembled the natural enamel structure. In this investigation, OP values exhibited significant variations among material layers and were diminished when glass was applied, and a faster cooling strategy was implemented. The OP values in the study ranged from 1.39 to 2.25, which were lower than those observed in human enamel (19.8 - 27.6) (13). This difference might be related to the quantity of color additives, affecting the pathway of light through the materials and consequently altering the opalescence effect.

The color difference value is crucial for determining the amount of color alteration related to the VITA Classical shade tab, with a lower ΔEdiff value indicating less color alteration or better color stability. Among the material types, the C-zone of Mu had the lowest ΔEdiff value, indicating the least color alteration. Glass-infiltrated 5Y-PSZ exhibited a higher color alteration than non-infiltrated 5Y-PSZ. Regarding the cooling strategy, the fast cooling produced a higher color alteration than others. However, the ΔEdiff values for different materials, glass applications, or cooling strategies remained within an AT (ΔEdiff≤5.5) (14). Performing glass infiltration with a fast cooling strategy on an incisal layer resulted in the highest ΔEdiff value, exceeding the acceptability limit (ΔEdiff>5.5) (14). According to the infiltration zone, slower cooling appeared to result in a shallower infiltration depth and thicker residual glass. This might be attributed to the limited capability of glass infiltration in porous zirconia. The porous zirconia subjected to slow-speed firing exhibited stronger grain integration with smaller and fewer gaps, which was inefficient for deep glass infiltration. However, the complete integration between the grain boundary and glass in slow cooling might have a more significant impact on enhanced translucency than the depth of infiltration.

Regarding the limitations of this study, distinguishing between the t’- and c-phases of zirconia using XRD was challenging due to their similar crystal structures and lattice parameters, resulting in strong overlapping diffraction patterns. In some cases, discrimination between the two phases can be achieved by assessing their tetragonality (c/a ratios) or utilizing alternative analytical techniques such as transmission electron microscopy (TEM) or Raman spectroscopy (30). Therefore, it is crucial to employ multiple analytical techniques to accurately identify and quantify the different phases present in a zirconia sample. Different zirconia brands, varying glass compositions and techniques, as well as the oral cavity environment, may yield different results, necessitating more in-depth investigation. Moreover, a study on the mechanical characteristics of 5Y-PSZ with the devised glass infiltration and cooling strategies should be conducted to extend the application of these materials in high occlusal load areas.

Based on this recent study, the color parameters of 5Y-PSZ were influenced by the type of materials, glass infiltration, and cooling speed, except for the CR value, which was not influenced by material type. The translucency and opalescence of Mo were the highest, followed by MuC and MuI 5Y-PSZ. Glass infiltration reduced translucency and opalescence, but increased contrast and color alteration due to the differences in refractive indices among various compositions. The fast cooling strategy also decreased translucency and opalescence but increased contrast and color alteration since it interrupted the complete integration of grains and produced smaller grains, leading to more light scattering. The application of glass infiltration and increasing the cooling rate resulted in a whiter appearance, less red-yellow, and more green-blue in 5Y-PSZ. However, the perception of color alteration was within an acceptable threshold (ΔEdiff≤5.5).

## Conclusions

Materials, glass infiltration, and cooling strategies, along with their interactions altered the color characteristics of 5Y-PSZ, except for material to CR, the interaction of material and cooling to CR, and the interaction of glass and cooling to TP and CR. The monochrome 5Y-PSZ was more translucent than the multilayer 5Y-PSZ, possibly due to pigment additives. Glass infiltration decreased translucency and opalescence, but increased contrast and color alteration, probably due to the different refractive indices of components. Accelerating the cooling speed of 5Y-PSZ resulted in lower translucency and opalescence, but greater contrast and color alteration as it produced smaller grains and a transformation from t- to m-phase. The application of glass and increasing the cooling rate produced a whiter appearance, reduced red-yellow, and increased green-blue in 5Y-PSZ. However, the color alteration for different materials, glass applications, or cooling strategies remained within an accepTable limit.

Clinical implications

To produce materials with enhanced translucency, opalescence, and optimal color alteration, it is recommended to use monochrome 5Y-PSZ, combined with slow cooling, without glass infiltration. The glass-infiltrated zirconia produces a glossy surface compared to its non-infiltrated counterpart. However, the application of glass and accelerating the cooling rate decreased translucency and opalescence, but increased contrast and color alteration. Nevertheless, it could be deemed accepTable for color alteration, meaning accepTable color stability upon infiltration with glass and raising sinter cooling rate. Thus, glass infiltration sintering and accelerated cooling rate were suggested for the benefit of cost-effective restoration fabrication from 5Y-PSZ.

## Figures and Tables

**Table 1:** Material, abbreviation (Abv.), brand, manufacturers, batch number, and composition (weight %) of materials used in this study.

Material	Abv.	Brand	Manufacturer	Batch no.	Composition (weight %)
Monochrome 5mol% yttria-partially stabilized zirconia	Mo	Cercon xt	Dentsply Sirona, Charlotte, NC, USA	18040682	≥99% ZrO_2_+HfO_2_+Y_2_O_3_, 9% Y_2_O_3, _<3% HfO_2 _, <1% Al_2_O_3,_+SiO_2_
Multilayer 5mol% yttria-partially stabilized zirconia	Mu	Cercon xt ML	Dentsply Sirona, Charlotte, NC, USA	18041981, 18042302	≥99% ZrO_2_+HfO_2_+Y_2_O_3, _9% Y_2_O_3, _<3% HfO_2 _, <1% Al_2_O_3,_+SiO_2_
Glass infiltration powder	G	In-house glass	Division of Biomaterials, Khon Kaen University, Khon Kaen, Thailand	202201	66.8% SiO_2_, 12% Al_2_O_3_, 10% Na_2_O, 7.9% K_2_O, 3% CaO, 0.3% Fe+Ti+Mg

**Table 2:** Mean, standard deviation (SD) of translucency parameter (TP), contrast ratio (CR), opalescence parameter (OP), color difference (∆Ediff), relative monoclinic (m-), tetragonal (t-), and cubic (c-) phase content (wt.%), and percentage of ultrafine (u), fine (f), medium (m), and large (l) grain size distribution (%) of of traditional (T) and glass infiltrated (G), monochrome (Mo), multilayer [(Mu: cervical (C), incisal (I)] 5Y-PSZ upon slow- (S), normal- (N), and fast- (F) cooling rates.

Group	n	TP	CR	OP	∆E_diff_	Phase (wt%)	Grain size (%)
		Mean±SD	Mean±SD	Mean±SD	Mean±SD	m- / t- / c-	U / f / m / l
TMoS	15	2.8±0.1	0.94±0.01	2.2±0.1	2.8±1.5	4.4 / 49.8 / 45.8	0.0 / 16.4 / 35.8 / 47.8
TMuCS	15	2.6±0.1	0.94±0.01	2.0±0.1	1.9±0.3	4.9 / 50.1 / 45.0	1.5 / 12.1 / 39.4 / 47.0
TMuIS	15	2.4±0.1	0.94±0.01	1.7±0.1	4.4±0.3	4.5 / 50.5 / 45.0	0.0 / 18.3 / 35.4 / 46.3
TMoN	15	2.8±0.1	0.94±0.01	2.2±0.1	3.1±1.4	4.4 / 50.0 / 45.6	1.3 / 21.3 / 29.3 / 48.0
TMuCN	15	2.6±0.1	0.94±0.01	2.0±0.1	3.1±0.5	5.1 / 49.4 / 45.5	0.0 / 19.7 / 31.6 / 48.7
TMuIN	15	2.4±0.1	0.94±0.01	1.7±0.1	5.3±0.4	5.2 / 50.3 / 44.5	4.6 / 24.1 / 25.0 / 46.3
TMoF	15	2.6±0.2	0.94±0.01	2.1±0.1	2.7±0.6	5.0 / 49.3 / 45.7	8.1 / 27.6 / 19.5 / 44.8
TMuCF	15	2.5±0.1	0.94±0.01	1.9±0.1	2.4±0.5	6.0 / 50.4 / 43.6	0.0 / 29.7 / 30.7 / 39.6
TMuIF	15	2.2±0.1	0.95±0.01	1.6±0.1	4.4±0.3	5.4 / 50.4 / 44.2	1.4 / 16.7 / 43.1 / 38.9
GMoS	15	2.4±0.2	0.96±0.02	2.0±0.2	4.8±0.2	5.5 / 49.6 / 44.9	2.3 / 10.2 / 21.6 / 65.9
GMuCS	15	2.2±0.2	0.96±0.01	1.9±0.1	4.4±0.4	6.4 / 49.9 / 43.7	4.1 / 8.1 / 16.2 / 71.6
GMuIS	15	2.1±0.2	0.96±0.02	1.7±0.2	5.9±0.6	6.6 / 52.0 / 41.4	2.4 / 7.1 / 16.7 / 73.8
GMoN	15	2.2±0.1	0.96±0.02	1.9±0.2	5.4±0.3	5.4 / 47.1 / 47.5	2.3 / 7.9 / 14.6 / 75.3
GMuCN	15	2.3±0.2	0.95±0.01	1.7±0.1	5.6±0.6	5.2 / 48.8 / 46.0	2.7 / 12.0 / 24.0 / 61.3
GMuIN	15	2.2±0.2	0.95±0.01	1.8±0.1	5.4±0.6	5.2 / 50.5 / 44.3	0.0 / 14.0 / 5.6 / 60.5
GMoF	15	2.1±0.2	0.96±0.02	1.8±0.1	5.1±0.4	7.3 / 49.5 / 43.2	0.0 / 10.6 / 27.7 / 61.7
GMuCF	15	2.0±0.2	0.96±0.01	1.6±0.2	5.4±0.7	5.7 / 47.6 / 46.7	0.0 / 14.0 / 27.9 / 58.1
GMuIF	15	2.0±0.1	0.95±0.01	1.4±0.1	7.3±0.2	6.3 / 49.7 / 44.0	3.0 / 13.1 / 19.2 / 64.7

**Table 3:** Three-way ANOVA of (a) translucency parameter (TP), (b) contrast ratio (CR), (c) opalescence parameter (OP), (d) color difference (∆Ediff), of traditional and glass infiltrated sintering of monochrome and multilayer 5Y-PSZ with different cooling protocols.

(a) ANOVA of TP upon different factors					
Source	SS	df	MS	F	p
Material	3.492	2	1.746	78.253	0.001
Sintering	9.057	1	9.057	405.952	0.001
Cooling	2.537	2	1.269	56.867	0.001
Material * Sintering	1.125	2	.562	25.206	0.001
Material * Cooling	.276	4	.069	3.091	0.017
Sintering * Cooling	.079	2	.040	1.780	0.171
Material * Sintering * Cooling	.271	4	.068	3.041	0.018
Error	5.622	252	.022		
(b) ANOVA of CR upon different factors					
Material	.000	2	.000	1.413	0.245
Sintering	.020	1	.020	165.382	0.001
Cooling	.002	2	.001	7.554	0.001
Material * Sintering	.001	2	.001	4.580	0.011
Material * Cooling	.001	4	.000	1.413	0.230
Sintering * Cooling	.000	2	.000	2.032	0.133
Material * Sintering * Cooling	.001	4	.000	2.763	0.028
Error	.030	252	.000		
(c) ANOVA of OP upon different factors					
Material	6.570	2	3.285	199.021	0.001
Sintering	2.112	1	2.112	127.978	0.001
Cooling	1.944	2	.972	58.896	0.001
Material * Sintering	.789	2	.395	23.903	0.001
Material * Cooling	.210	4	.053	3.188	0.014
Sintering * Cooling	.268	2	.134	8.104	0.001
Material * Sintering * Cooling	.279	4	.070	4.231	0.002
Error	4.159	252	.017		
(d) ANOVA ∆E_diff_ upon different factors					
Material	143.491	2	71.745	172.209	0.001
Sintering	303.308	1	303.308	728.025	0.001
Cooling	19.913	2	9.956	23.898	0.001
Material * Sintering	17.328	2	8.664	20.796	0.001
Material * Cooling	14.470	4	3.618	8.683	0.001
Sintering * Cooling	15.437	2	7.719	18.527	0.001
Material * Sintering * Cooling	15.179	4	3.795	9.109	0.001
Error	104.988	252	.417		

NB: SS: sum of squares, df: degree of freedom, MS: mean square, F: F-ratio.

**Table 4:** Post hoc Bonferroni multiple comparisons of (a) translucency parameter (TP), (b) contrast ratio (CR), (c) opalescence parameter (OP), (d) color difference (∆Ediff) of traditional (T) and glass infiltrated (G) monochrome (Mo) and multilayer [Mu, cervical (C), incisal (I)] 5Y-PSZ upon slow (S), normal (N), and fast (F) cooling protocols.

(a) Post hoc of TP as a function of material, sintering, and cooling protocol										
Material	Mo	MuC	MuI	Sintering	T	G	Cooling	S	N	F
Mo	1	0.001	0.001	T	1	0.001	S	1	1	0.001
MuC		1	0.001	G		1	N		1	0.001
MuI			1				F			1
(b) Post hoc of CR as a function of material, sintering, and cooling protocol										
Material	Mo	MuC	MuI	Sintering	T	G	Cooling	S	N	F
Mo	1	0.490	0.399	T	1	0.001	S	1	0.073	0.331
MuC		1	1	G		1	N		1	0.001
MuI			1				F			1
(c) Post hoc of OP as a functionof material types, glass infiltration and cooling protocols										
Material	Mo	MuC	MuI	Sintering	T	G	Cooling	S	N	F
Mo	1	0.001	0.001	T	1	0.001	S	1	0.001	0.001
MuC		1	0.001	G		1	N		1	0.001
MuI			1				F			1
(d) Post hoc of ∆E_diff_ as a function of material, sintering and cooling protocol										
Material	Mo	MuC	MuI	Sintering	T	G	Cooling	S	N	F
Mo	1	0.125	0.001	T	1	0.001	S	1	0.001	0.001
MuC		1	0.001	G		1	N		1	0.951
MuI			1				F			1

## Data Availability

The datasets used and/or analyzed during the current study are available from the corresponding author.
